# Pharmacist-Led Chronic Care Management for Medically Underserved Rural Populations in Florida During the COVID-19 Pandemic

**DOI:** 10.5888/pcd17.200265

**Published:** 2020-07-30

**Authors:** Madison Como, Chenita White Carter, Margareth Larose-Pierre, Kellie O’Dare, Cynthia R. Hall, Jason Mobley, Gervin Robertson, Jason Leonard, Lindsey Tew

**Affiliations:** 1 Florida Agricultural and Mechanical University College of Pharmacy and Pharmaceutical Sciences, Institute of Public Health, Tallahassee, Florida

## Abstract

Medically underserved patients in rural areas are more vulnerable to poor health outcomes, including the risks associated with coronavirus disease 2019 (COVID-19). Pharmacists, student pharmacists, and other health care professionals are working together to implement new, innovative ways to deliver the same standard of care during the COVID-19 pandemic to these vulnerable patients. These services include telehealth with virtual and telephone medication therapy management sessions led by ambulatory care pharmacists and student pharmacists. Pharmacists, student pharmacists, and other health care professionals should continue to adapt to these new technologies to improve health outcomes for their patients during the pandemic.

**Summary**

**What is already known on this topic?**

Ambulatory care pharmacists play an important role in chronic care and medication therapy management. These pharmacists and student pharmacists work closely with health care providers to deliver medication-related recommendations.

**What is added by this report?**

We discuss how 2 pharmacist faculty members from Florida Agricultural and Mechanical University College of Pharmacy and Pharmaceutical Sciences, Institute of Public Health are using telehealth during the coronavirus disease 2019 (COVID-19) pandemic to continue to care for patients in an ambulatory care setting as well as student pharmacists’ contributions.

**What are the implications for public health practice?**

With the implementation of telehealth services, patients can continue receiving care from our ambulatory care pharmacists and student pharmacists to help improve health-related outcomes.

## Introduction

According to the World Health Organization (WHO), the novel coronavirus, severe acute respiratory syndrome coronavirus 2 (SARS-CoV-2; the cause of coronavirus disease 2019 [COVID-19]), first isolated in China, has caused over 9,473,214 confirmed cases worldwide and over 484,249 confirmed deaths (1). COVID-19 officially spread to the United States with the first confirmed case on January 20, 2020 (2). The Centers for Disease Control and Prevention state that the signs and symptoms associated with the virus include cough, shortness of breath or difficulty breathing, fever, chills, muscle pain, sore throat, and loss of taste or smell (3). During the pandemic, many people have become more anxious about acquiring the virus. People in the United States have been instructed to practice self-isolation and social distancing to help prevent the spread of COVID-19. This presents an opportunity for health care professionals, including pharmacists, public health providers, and students to assist with the health care needs of people living in various geographical locations across the country. In this commentary, we share information on what Florida Agricultural and Mechanical University College of Pharmacy and Pharmaceutical Sciences, Institute of Public Health (FAMU CoPPS, IPH) is doing to continue to sustain patient care services and support to population health in rural northwest Florida.

Northwest Florida has a large rural and medically underserved population, with much of the area being a designated Health Professional Shortage Area (HPSA) (4). Many of the patients in northwest Florida have limited access to medical care for various reasons. One of the community health centers in the Pensacola area is a Federally Qualified Health Center (FQHC) that focuses largely on serving the medically underserved population. Most patients (59%) seen at the clinic reported insurance through Medicaid, 20% reported having no insurance, 13% reported private insurance, and the remaining 8% reported insurance with Medicare (5). The clinic serves patients regardless of demographics, including White, African American, Asian, multiracial, and other races. Some of the patients served by the clinic are homeless, uninsured, non-English speakers, veterans, and school-based health center patients. In 2019, the clinic had 133,974 medical visits, with 9,944 patients having hypertension, 3,017 with asthma or chronic obstructive pulmonary disease, 3,873 with diabetes, 17,441 with obesity, and 7,559 with anxiety, depression, or other mood disorders (5). Many of these patients receive chronic care, medication therapy management (MTM) services, or both.

## Chronic Disease Care Management

Patients with chronic diseases are at a higher risk of acquiring COVID-19 and suffering sequelae from the virus (6). Ambulatory care pharmacists play a vital role in chronic care management as well as MTM in the ambulatory care setting. Ambulatory care pharmacists, student pharmacists under their supervision, and other health care professionals work closely with patients who have various complex clinical chronic conditions (eg, diabetes mellitus, hypertension, dyslipidemia) that can present with diverse clinical symptomatology.

FAMU CoPPS, IPH has 2 ambulatory care pharmacist faculty members who work at the FQHC clinic in Pensacola, Florida. Each pharmacist has a cohort of patients to which they provide medication-related recommendations and counseling to help improve patient outcomes by using evidence-based guidelines (7,8). These pharmacists have also established an MTM program that focuses on cardiovascular disease and stroke prevention among the medically underserved population. In the MTM program, student pharmacists and pharmacists perform chart reviews, assess laboratory values, identify barriers to medication adherence, provide both pharmacologic and nonpharmacologic counseling, and make medication-related recommendations to referring providers. During each MTM interview, patients are asked about their health literacy level surrounding their medications. The goal of this exercise is to ensure that patients will become more comfortable, educated, and empowered with consistent MTM appointments, which will, in turn, improve medication adherence and health outcomes. The health outcomes being monitored for improvement include blood pressure, blood glucose, glycated hemoglobin A_1c_, and cholesterol levels.

Telehealth is becoming increasingly popular in health care settings, and ambulatory care pharmacy is no exception (9,10). With the implementation of telehealth services for patients, our ambulatory care pharmacists are able to continue to provide the same standard of care as they would during in-person encounters. Many patients are open to this new form of communication, especially those with transportation limitations or time constraints. Telehealth sessions allow patients to speak with the pharmacists from the comfort of their home. The FQHC clinic, along with other primary care clinics, has limited patient appointments and provider availability because of COVID-19, making the ambulatory care pharmacist’s role even more critical than before. With telehealth, our pharmacists can conduct patient interviews, provide medication counseling, and make medication-related recommendations to health care providers. Patients having continuous access to our pharmacists via telehealth can help improve medication adherence, safety, and patient clinical outcomes (11).

During the COVID-19 pandemic, it became essential to design the telehealth encounter with the appropriate technology to deliver a positive patient experience. The provision of iPads (Apple Inc) to patients and laptops to health care professionals that are preloaded with Zoom software (Zoom Video Communications, Inc) allows the patient a frictionless telehealth experience. The patient is only required to connect the device to their Wi-Fi network. If a patient does not have access to a Wi-Fi network, patient interviews can also be conducted via telephone.

The practice of health care professions is primarily governed by state law. Additionally, the US Department of Health and Human Services Office for Civil Rights (OCR) provided guidance that, during the COVID-19 pandemic, the office will not impose penalties for noncompliance with Health Insurance Portability and Accountability Act (HIPAA) provisions when telehealth is provided in “good faith” by using remote nonpublic-facing communication technologies such as Zoom (12). Thus, Zoom telehealth encounters with patients at the FQHC by providers is acceptable and appropriate.

## Educating Patients and the Public

As health care professionals, pharmacists are at the forefront of the pandemic in providing testing and educating the public about the virus, the tests used to detect the virus and antibodies, and the different treatment options for symptoms associated with the virus. Our ambulatory care pharmacists are relied upon to provide accurate information regarding all aspects of their duties such as the distribution of medications, tests, and medical devices; clinical services; and the education of their patients, caregivers, health care professionals, students, and other associates. During COVID-19, our pharmacists’ role has expanded beyond the previously multifaceted tasks. Our 2 ambulatory care pharmacists and student pharmacists are responsible for educating patients and the public regarding the proper use and safety of the medications being prescribed for COVID-19. According to the American Society of Health System Pharmacists, one of the pharmacists’ roles is to prevent diseases through vaccination (13). Several vaccines are being studied for COVID-19. Although those vaccines are not available, our pharmacists are certified to administer vaccines for other diseases. The COVID-19 pandemic offers tremendous opportunities for our pharmacists to prepare student pharmacists and educate health care providers and others about the virus, current treatments, and vaccines that are in the pipeline. Our pharmacists and student pharmacists are prepared to provide immunization against COVID-19 as they do for other vaccine-preventable diseases.

Our pharmacists also take every opportunity to educate the public about the virus and testing, treatment, and prevention of COVID-19 through flyers, webinars, patient counseling, emails, posters, in-service sessions, television, public service announcements, newsletters, public events, and health fairs (14). Our pharmacists also educate other health care professionals by providing information regarding vaccines and providing reminders for immunization dates, local immunization rates, and vaccines needed for their patients (15). Our pharmacists will continue to do so with COVID-19 vaccines in addition to promoting their appropriate use, and will participate in shared registries through the Florida Department of Health.

## Student Pharmacist Involvement

Student pharmacists are working alongside pharmacists and participating in many ways to help with COVID-19–related efforts. Student pharmacists on advanced pharmacy practice experience rotations at the FQHC clinic are assisting pharmacists in interviewing patients and providing medication-related recommendations via telehealth under a pharmacist’s supervision. The national shortage of personal protective equipment (PPE) has caused many institutions, including the FQHC clinic, to limit student interaction on site for rotations, so alternative ways to use students to combat COVID-19 have been implemented. Student pharmacists assisting with telehealth patient interviews require no PPE and have minimal COVID-19 exposure risk to their patients and others. These students have the full experience of interviewing patients, formulating care plans, and making recommendations to health care providers via platforms such as Zoom or telephone consultation.

In addition to providing telehealth services, student pharmacists on advanced pharmacy practice experience rotations at another clinic for the medically underserved in the Pensacola area are assisting with curbside prescription pickup and counseling sessions. These students are required to wear PPE and practice social distancing while educating patients about their medications. This opportunity allows the students to gain experience and confidence in conveying pharmaceutical care by applying their knowledge and skills and gaining professional competence.

## Training and Development of Future Pharmacists

The COVID-19 pandemic is an opportunity for colleges and schools of pharmacy to prepare student pharmacists before sending them into the workforce. Student pharmacists at FAMU CoPPS, IPH are being trained to administer vaccines and must complete their immunization training as part of their graduation requirements. Training for student pharmacists and pharmacists to administer vaccines requires an in-person component, but the bulk of the learning and training can be done by using virtual interactions and live or prerecorded webinars during the pandemic. Student pharmacists are also learning how to educate patients, caregivers, health care providers, and the public about COVID-19 and its treatment and prevention.

## Implications for Public Health

The COVID-19 pandemic has greatly affected many communities, especially medically underserved rural populations (16). Telehealth can address both proximity and cultural barriers by effectively increasing access and improving outcomes in rural areas (17). Medically underserved populations in northwest Florida have experienced a lack of medical care because of social distancing measures and limited provider accessibility during the COVID-19 pandemic. With telehealth services, these patients still have access to ambulatory care pharmacists regardless of their ability to make traditional in-person clinic appointments. Medically underserved rural populations have been able to continue receiving care through the delivery of various telehealth services. Some of these provisions include virtual or telephone patient encounters with ambulatory care pharmacists and student pharmacists. It is imperative that pharmacists, student pharmacists, and other health care professionals continue to adapt to these new technologies and familiarize themselves with the laws governing their practice to deliver the same standard of care to improve health outcomes for their patients during the pandemic.
